# Towards a mathematical understanding of invasion resistance in multispecies communities

**DOI:** 10.1098/rsos.231034

**Published:** 2023-11-15

**Authors:** Erida Gjini, Sten Madec

**Affiliations:** ^1^ Center for Computational and Stochastic Mathematics, Instituto Superior Tecnico, Lisbon, Portugal; ^2^ Laboratory of Mathematics, University of Tours, Tours, France

**Keywords:** colonization resistance, microbial ecology, multispecies community, pairwise invasion fitness matrix, replicator equation, system invasibility

## Abstract

Multispecies community composition and dynamics are key to health and disease across biological systems, a prominent example being microbial ecosystems. Explaining the forces that govern diversity and resilience in the microbial consortia making up our body’s defences remains a challenge. In this, theoretical models are crucial, to bridge the gap between species dynamics and underlying mechanisms and to develop analytic insight. Here we propose a replicator equation framework to model multispecies dynamics where an explicit notion of invasion resistance of a system emerges and can be studied explicitly. For illustration, we derive the conceptual link between such replicator equation and *N* microbial species’ growth and interaction traits, stemming from micro-scale environmental modification. Within this replicator framework, mean invasion fitness arises, evolves dynamically, and may undergo critical predictable shifts with global environmental changes. This mathematical approach clarifies the key role of this resident system trait for invader success, and highlights interaction principles among *N* species that optimize their collective resistance to invasion. We propose this model based on the replicator equation as a powerful new avenue to study, test and validate mechanisms of invasion resistance and colonization in multispecies microbial ecosystems and beyond.

## 1. Introduction

Deciphering the rules and mechanisms governing dynamics of living systems is a daunting task. Theoretical models contribute to meet this challenge, as they provide formalisms for precise quantitative descriptions of phenomena, clear hypotheses formulation and cause-and-effect predictions within a set of pre-stated assumptions. One of the very widely used models in ecology today is the Lotka–Volterra modelling framework for *N* species, which abstractly describes how the abundances of a set of species change with time as a function of their growth rates and mass-action interaction parameters, typically describing competition [1–3]. In this model, the reproductive rate (fitness) of each species depends linearly on the abundance of other species. This model has a long history of study, refinement and applications in many areas of biology and ecology [3–7].

Another classical model originating from evolutionary game theory is the replicator equation formalism [8], tracking the relative evolution of *N* competing strategies, and how they spread in a population, and is mostly used in population genetics, economics, theoretical physics and evolutionary dynamics. Although it is known that the replicator equation is closely related to Lotka–Volterra model (topologically equivalent, to be mathematically strict) [9,10], most models of multispecies dynamics utilize the Lotka–Volterra framework, while applications of the replicator equation in ecology remain rarer.

Here, we propose a replicator equation framework to model multispecies dynamics where species frequencies can be tracked explicitly from the *N* variables of the system. The key aspect of evolutionary game theory, explicit in the replicator equation, is frequency-dependent selection, whereby the fitness of an individual depends on the frequency of other ‘strategies’ in the population. We take advantage of this explicit nature to highlight that furthermore, within the replicator equation framework, an explicit notion of invasion resistance of the system emerges naturally as a term in the equation and can be studied. In contrast, it is not natural to see mean invasion fitness of a system explicit in a typical Lotka–Volterra framework.

Invasion resistance is important in all ecosystems [3,4,11,12], and the replicator framework is flexible to be applied to many modelling contexts, but in the following, without loss of generality, we will first elaborate an illustration focused on a polymorphic microbial ecosystem, and then synthesize some broader qualitative insights for multi-species invasion resistance under replicator dynamics and various special cases.

*From empirical colonization resistance to mathematical invasion resistance*. A pertinent example of a microbial ecosystem where invasion, interaction and colonization processes among several species are important is the commensal microbiota, which plays a crucial role in health and disease. Recent studies are increasingly uncovering one of its clearest contributions, across organisms: protection against invading pathogens, known as colonization resistance [13,14]. In humans, this protection is important when enteric bacterial pathogens challenge the gastrointestinal tract, but applies also in the nasopharynx, skin, and other environments which are populated by rich consortia of commensal microbes. The composition of gut microbiota affects several collective metabolic functions and feedbacks with the host’s immune system, leading to or sometimes impairing immune homeostasis. Disruption of healthy microbiota (dysbiosis), for example through antibiotics, or cytotoxic chemotherapy, can result in loss of colonization resistance and increased susceptibility to pathogens [15,16], as well as long-term shifts in microbial ecosystem composition [17] and other diseases [18]. Conversely, reconstitution of normal microbiota (e.g. through faecal transplants) has been demonstrated to restore host protection, and help cure patients from recurring pathogenic infections [19]. Such dynamic processes of loss and gain of colonization resistance are particularly important in the intestinal tract, making stability, diversity and resilience of our gut microbial ecosystem an intense topic of investigation [14,20–22].

Despite the progress in experimentally revealing the beneficial roles of the greater than 100 species (trillions of microbes) populating the human intestine [23], understanding the microbiota from a quantitative perspective is a hugely difficult challenge with difficulties in collecting useful data, and then identifying scalable mathematical models to tackle what can quickly become incredibly complex highly dimensional problems. Within this highly complex and fast-growing research field, a theoretical understanding of how colonization resistance emerges as a collective trait from a community network, is maintained as a dynamic process through host lifespan, and reacts to various perturbations remains elusive. With this study, we hope to provide some new tools and concepts to meet this challenge.

In particular, to contribute to this question, we synthesize a new conceptual and mathematical perspective to describe colonization resistance at the level of a single host, based on the mathematical quantity of mean invasion fitness emerging from a replicator equation system for *N* species.

## 2. Modelling invasion resistance based on replicator dynamics

The classical *N*-dimensional replicator equation [8] can be written as follows:

2.1 $$\frac{dz_{i}}{d\tau }=z_{i}(f_{i}(z)-\bar{\;f}(z)),\;i=1,\ldots ,N$$

where **z** = (*z*_*i*_)_*i*=1, …,*N*_ are the frequencies of *N* competing strategies in a population with $\sum_{i}z_{i}=1$, *f*_*i*_(**z**) is the fitness of strategy *i* and $\bar{\;f}(z)=\sum_{i=1}^{N}z_{i}f_{i}(z)$ the mean fitness over all strategies in the population. Of course, *f*_*i*_ may be time- and system-dependent. In the most general form of this equation, there are no constraints on how linear or nonlinear *f*_*i*_(**z**) is as a function of **z**. The equations describe how the frequencies *z*_*i*_ change over time as a function of other strategies in the population; essentially, one strategy will go up in success at any point in time if its fitness is higher than the mean fitness of all strategies present in the system.

Here, we propose the following special version of the replicator equation, with a linear fitness function, to model explicit *N*-species frequency dynamics:

2.2 $$\frac{dz_{i}}{d\tau }=\Theta z_{i}\left(\sum_{\;j\neq i}\lambda_{i}^{\;j}z_{\;j}-\underset{1\leq k\neq j\leq N}{\sum \sum }\lambda_{k}^{\;j}z_{\;j}z_{k}\right),\;i=1,\ldots ,N$$

with $\sum_{i}z_{i}=1$. In our formulation, $\lambda_{i}^{\;j}$ denote pairwise invasion fitnesses between species *i* and *j* and Θ is a speed constant (typically assumed 1 in the classical replicator equation). Hence in this replicator equation, $f_{i}(z)=\sum_{\;j\neq i}\lambda_{i}^{\;j}z_{j}$ and $\bar{\;f}(z)$ is the quadratic term $Q=\sum_{1\leq k,j\leq N}\lambda_{k}^{\;j}z_{\;j}z_{k}$. The mutual invasion fitness, appearing in this fitness function, $\lambda_{i}^{\;j}$, is a key quantity at the heart of adaptive dynamics [24], and depicts the initial demographic performance, namely the invasion growth rate of a species *i* in an equilibrium set by a single resident *j*. Naturally, it is defined only for two distinct species, with $\lambda_{k}^{k}=0$.

The first feature to remark is that pairwise invasion fitness is typically derived from the underlying ecology of the model or details of the competition process between species. The second feature to remark is that every abstract replicator system with a linear payoff matrix *A*, through basic algebraic operations, can be brought into the special form (2.2) where the new payoff matrix *A*′ has zeros on the diagonal, a transformation that preserves exactly the dynamics, and in addition benefits from the extra attribute that the resulting payoff matrix could be read as a matrix of pairwise invasion fitnesses between the competing entities.

### 2.1. An emergent exact definition of invasion resistance

In this replicator equation (equation (2.2)), from the very meaning of the variables and the parameters, the dynamic quadratic term $Q=\sum_{1\leq k,j\leq N}\lambda_{k}^{\;j}z_{\;j}z_{k}$ (representing $\bar{\;f}(z)$ in equation (2.1)), equals precisely mean invasion fitness of the system. Indeed, noting that $\lambda_{k}^{k}=0$ and ${\bar{\lambda }}_{k}=\sum_{\;j=1}^{N}\lambda_{k}^{\;j}z_{j}$, we have

$$Q(z)=\sum_{k=1}^{N}\bar{\lambda_{k}}z_{k},$$

which reduces to

2.3 $$Q(z)\equiv \bar{\lambda }.$$

In other words, *Q* sums the global effect of the system on each member, and reflects mathematically whether the multispecies consortium as a whole adds (*Q* < 0) or detracts from the growth of each individual member (*Q* > 0) [25]. For any entity *i* in the system, the rate of growth of its frequency (*z*_*i*_) relative to other entities is ultimately given by $\bar{\lambda_{i}}-\bar{\lambda }$, namely by how it performs in pairwise invasion ‘games’ with any other member of the system, and also by how the entire system as a whole ($\bar{\lambda }$) acts to promote or reduce net individual growth. By the same arguments, *Q*(**z**) is also a quantity that explicitly embodies the invasion resistance of the system towards outsiders.

### 2.2. Invasion by outsiders depends on systemic invasion resistance

Mathematically, it is straightforward to see that the initial growth rate of any invader in a system at state **z**(*τ*) is given by

2.4 $$r_{\text{invader}}=\sum_{\;j\in \text{system}}\lambda_{\text{invader}}^{\;j}z_{j}-Q(z).\;$$

The first summation term in *r*_invader_ depends on invader traits, $\lambda_{\text{invader}}^{\;j}$, namely how this species invades any existing species currently in the system. The second term instead, −*Q*, is independent of the invader, and crucially describes how the system invasion fitness by itself detracts from or adds to invader’s initial growth, if *Q* > 0 or *Q* < 0, respectively. One can thus immediately use *Q* as a global indicator of systemic resistance to invasion by outsider strains, that applies at any point in time, independent of equilibrium or non-equilibrium dynamics.

### 2.3. Matching the generalized Lotka–Volterra and replicator equation framework

It is a well-known result that every generalized Lotka–Volterra system of dimension *n* − 1 (*y*_*i*_, *i* = 1, …, *n* − 1 with $y=\sum_{i=1}^{n-1}y_{i}$) can be written as a replicator equation of dimension *n* (*z*_*i*_, *i* = 1, …*n*). The equivalence can be shown by the transformation *z*_*i*_ = *y*_*i*_/(*y* + 1) (*i* = 1, …, *n* − 1) and *z*_*n*_ = 1/(*y* + 1) [8]. Although the two models are topologically equivalent, they are not identical [8,9], and it has been argued that sometimes one formalism can be preferred over the other. One or the other formalism may be more easily connected to data, may be more easily amenable to analysis and hard proofs, or sometimes it may be a matter of convenience, practicality and personal conceptual choice of the modeller for the system of study. In our perspective, the above-mentioned mathematical link is the most important one for bridging between our work here using the replicator equation and the studies based on generalized Lotka–Volterra modelling of multispecies dynamics.

While discussions are old and ongoing in the literature about the merits and limitations of the Lotka–Volterra approach [26–29], one of which being the lack of explicit characterization of the underlying limiting resource in the system, further comparison between the Lotka–Volterra framework and the replicator equation falls beyond the scope of this paper, but see [8–10].

For us, it is natural to look at the replicator equation (2.2), how it arises by itself in an ecological context, noting that the key quantities modelled are not the same as those in the Lotka–Volterra equations, and the meaning of the parameters involved is not the same. Most crucially, invasion resistance of the system emerges naturally within the replicator equation, while it is not common to see mean invasion fitness of a system explicit in a typical Lotka–Volterra framework.

## 3. Invasion fitnesses from first principles and specific models

The pairwise invasion fitness coefficients are at the core of the replicator dynamics (equation (2.2)) and reflect initial demographic performance in dyadic *i* → *j* invasion scenarios between two species, starting from low frequency of *i* in an equilibrium set by *j* [24]. There are at least two key challenges regarding invasion fitnesses: (i) the first one is how to derive them bottom-up mechanistically from the details of the underlying ecology and biology of species dynamics and interactions; (ii) the second one is how to make strong qualitative and quantitative predictions about their effect on global dynamics once their structure and magnitudes are known.

In this paper, we address both of these challenges. First we show one possible route for the derivation of $\lambda_{i}^{\;j}$, bottom-up, exploiting an analogy with an SIS transmission dynamics framework proposed for multiple strains [25,30,31]. Application of this model to new contexts, for example, to in-host microbiota dynamics, requires re-interpretation of original variables and parameters, and a conceptual shift of scales. In §§3.1–3.3, we present the key ingredients for such re-interpretation, and develop the analogy for translation of the model to the within-host level.

Secondly, in §4, we illustrate how this model can be used and which predictions can be made based purely on knowledge of the Λ matrix structure between *N* species.

### 3.1. The analogy: from SIS to within-host microbial dynamics

**Figure 1.**
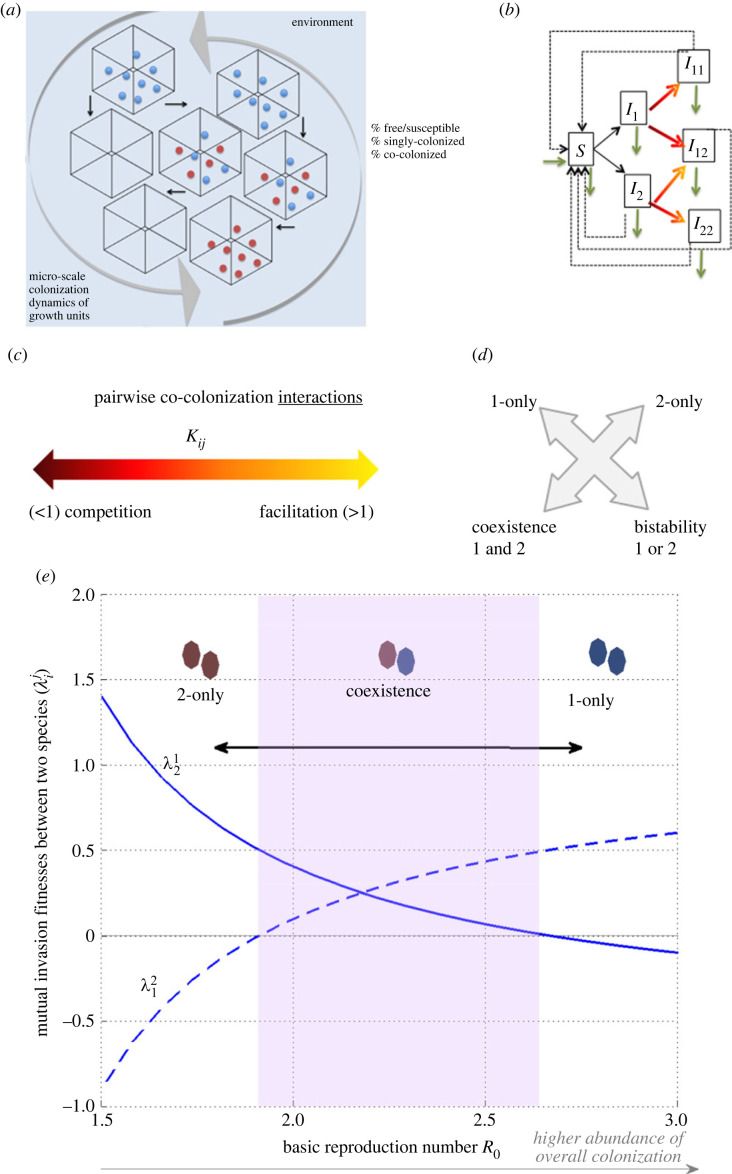
A conceptual analogy for within-host microbiota dynamics (here illustrated for *N* = 2). One mechanistic path to arrive at the replicator equation (equation (2.2)) is from the population structure model, based on epidemiological SIS dynamics with co-infection [25]. (*a*) Conceptualization of the within-host environment, where micro-compartments can be free (uncolonized *S*), singly colonized (*I*_*i*_) or doubly colonized (*I*_*ii*_, *I*_*ij*_, *I*_*jj*_) by microbes. Full equations are provided in §3.2. (*b*) Model structure for compartmental state transitions, including colonization (black arrows), co-colonization (colour-shaded arrows) and clearance (dashed arrows). Natural birth/death is denoted by green arrows. (*c*) Singly colonized compartments by one species (*i*) may have a reduced or increased susceptibility to becoming co-colonized by a second species (*j*), noted by coefficients *K*_*ij*_ below or above 1. For similar species, we may write: $K_{ij}=k+\epsilon \alpha_{ij}$, with the parameter 0<ϵ≪1 being small enough. (*d*) For *N* = 2, there are four outcomes (table 1), depending on species interactions: exclusion of 1 or 2, coexistence, or bistability. This is mathematically encoded in the mutual signs of the invasion fitnesses $\lambda_{i}^{\;j}$, whether (+ , −), (− , +), (+ , +) or (− , −). (*e*) Context-dependence of mutual invasion fitnesses, in this mechanistic model, where $\lambda_{i}^{\;j}=\alpha_{\;ji}-\alpha_{\;jj}-\frac{1}{k(R_{0}-1)}(\alpha_{ij}-\alpha_{\;ji})$. If *k* or *R*_0_ change, with *α*_*ij*_ fixed, the pairwise invasion fitnesses change and generate different ecology between 2 species. In this example, starting from coexistence (shaded region with both $\lambda_{1}^{2}$ and $\lambda_{2}^{1}$ positive), lowering overall colonization (*R*_0_ ↓ ), e.g. via antibiotics, or increasing it drastically, may lead to competitive exclusion of an opposite species in each direction. Going from stable coexistence of 2 species to competitive exclusion with a single winner reduces invasion resistance of the system from a positive value to 0. In an *N*-species network such shifts can be more complex but yet predictable [30].

*A host is a system of ‘free’ and ‘occupied’ micro-niches for colonization*. We define the host as a well-mixed system, and the dimensionality of the system as the number of similar microbial types (entities) interacting in colonization of such a system, hereafter denoted as species. In the re-framing following [25], we model the within-host environment as a number of potential micro-‘niches’ depicting generic growth units (resources) to be used, and we track the proportion of such niches that remain free or susceptible (*S*), those that get singly colonized by any species (*I*_*i*_), and those that get co-colonized, either twice by the same species (*I*_*ii*_) or by two different species (*I*_*ij*_) (see §3.2, and figure 1*a*). By taking suitably small such growth units within-host, we can truncate the multiplicity of ‘infection’ (MOI) to at most two co-colonizing species per unit. It is assumed that such growth units (similar to hosts in the epidemiological setting) homogeneously mix in the system, and when they are free, they are equally accessible to propagules of all species, whereas when they are already colonized, they become more or less available to co-colonization/co-utilization by others, depending on the identities of the interacting partners.

**Table 1.** Key features of the replicator dynamics emerging from a co-colonization model for similar species [25]. But see [31,39] for the model with 5-trait dimensions where species can vary.

*the constituent variables and parameters are the following:*	
micro-scale co-colonization interactions (*N* × *N*)	$K_{ij}=k+\epsilon \alpha_{ij}$ (similarity: 0≤ϵ≪1)
normalized interaction strengths	$\alpha_{ij}=(K_{ij}-k)/\epsilon$ (e.g. *k* = mean of *K*_*ij*_)
basic reproduction number	*R*_0_ = *β*/(*γ* + *r*)
proliferation/propagation and clearance rate	*β* and *γ*
replenishment of susceptible/free growth units	*r*
equilibrium colonization prevalence (I* + D*)	1 − S* = 1 − 1/*R*_0_
ratio of single to co-colonization (I*/D*)	*μ* = [(*R*_0_ − 1)*k*]^−1^
speed of multispecies dynamics	$\Theta =\beta (1-(1/R_{0}))(\mu /2{\left(\mu +1\right)}^{2}-\mu )$
timescale of selection (slow timescale)	τ=ϵt
frequency of species *i*	*z*_*i*_(*τ*), with $\sum z_{i}=1$, *i* = 1, …, *N*
single and co-colonization prevalence *i*/(*i*, *j*)	*I*_*i*_ = I*z_*i*_; *I*_*ij*_ = D*z_*i*_ *z*_*j*_
initial species composition and relative abundances	**z**(0)
trait-based mutual invasion fitness, in this model*:	
$\lambda_{i}^{\;j}=\alpha_{\;ji}-\alpha_{\;jj}-\mu (\alpha_{ij}-\alpha_{\;ji})$	
*frequency dynamics for N = 2 are given by:*	
$\frac{dz_{1}}{d\tau }=\Theta z_{1}\cdot [\lambda_{1}^{2}(1-z_{1})-(\lambda_{1}^{2}+\lambda_{2}^{1})z_{1}(1-z_{1})]$, *z*_2_ = 1 − *z*_1_	
(*N = 2*) *4 mutual invasion structures*	*four outcomes: types of edges* (*N species network*)
$\left(\lambda_{1}^{2},\lambda_{2}^{1})=(+,-\right)$ 1 ← 2	1 excludes 2: $z_{1}^{*}=1,z_{2}^{*}=0$
$\left(\lambda_{1}^{2},\lambda_{2}^{1})=(-,+\right)$ 1 → 2	2 excludes 1: $z_{1}^{*}=0,z_{2}^{*}=1$
$\left(\lambda_{1}^{2},\lambda_{2}^{1})=(+,+\right)$ 1 — 2	coexistence: $z_{1}^{*}=\frac{\lambda_{1}^{2}}{\lambda_{2}^{1}+\lambda_{1}^{2}},z_{2}^{*}=1-z_{1}^{*}$
$\left(\lambda_{1}^{2},\lambda_{2}^{1})=(-,-\right)$ 1 — 2	bistability: $z_{1}^{*}=1,$ or 0 depending on *z*_1_(0)
*environmental scenarios*	*effect on parameters* (…→Λ,Θ)
factors promoting colonization for all species	*R*_0_ ↑
increased microbial antagonism (or facilitation)	*k* ↓ (or *k* ↑)
different species number/composition	different *N*/*K*_*ij*_ entries
metabolic cross-feeding/bacteriocin effects	possibly different *K*_*ij*_, different *R*_0_
broad spectrum antibiotic treatment ↑ (↓)	*R*_0_ ↓ (*R*_0_ ↑)
different nutrient substrate/growth efficiency	different *β*
*the system with N interacting species evolves according to replicator dynamics:*	
$\frac{dz_{i}}{d\tau }=\Theta z_{i}\cdot \left(\sum_{\;j\neq i}\lambda_{i}^{\;j}z_{\;j}-{\sum \sum }_{1\leq k\neq j\leq N}\lambda_{\;j}^{k}z_{\;j}z_{k}\right),\;i=1,\ldots ,N$	
*colonization resistance as mean invasion fitness of the system*	
$Q(z)={\sum \sum }_{1\leq k\neq j\leq N}\lambda_{\;j}^{k}z_{\;j}z_{k}=\sum_{\;j}{\bar{\lambda }}_{\;j}z_{\;j}=\bar{\lambda }$	
*Q* captures the tension between individual and group success in the system	
— if *Q* > 0, the system as a whole acts negatively on each species, making *Q* a cost to any current member of the consortium, but by consequence, affecting negatively also invaders, thus a net benefit for system external stability	
— if *Q* < 0, the system as a whole acts positively on all species, promoting their growth, but also facilitating invaders	
— *Q* can be seen as a function of time (*τ*), of species composition and relative abundances (**z**), and of pairwise invasion coefficients (Λ matrix)	

Within each single growth unit, microbial propagules can grow, then upon release, can be transmitted to other units. Microbial propagation dynamics, thus, under homogeneous mixing of propagules in the in-host milieu, can be described, to a first-order approximation, through simple mass-action kinetics (figure 1*b*). The growth of each microbial species depends on their transmission rate *β* and frequency of niches emitting propagules of that species. The transmission parameter, *β*, in this analogy, can be taken as a net parameter, encapsulating the chain of events including local growth happening in the micro-niche of ‘origin’, followed by migration, and diffusion of infectious propagules in the system, multiplied by the effective probability of settling in other ‘destination’ niches. In its basic version, the model assumes *β* is equal for all *i*, yielding reproductive fairness in the system (but see [31] for a formal proof and generalization to a higher number of traits varying among relatively similar species).

Interactions among species happen upon co-colonization, where the colonizer (*i*) and co-colonizer (*j*) microbes may compete or facilitate each other, each ordered pair with its own coefficient *K*_*ij*_ (figure 1*c*). We do not specify the underlying explicit ecological mechanisms, which may be synergistic or antagonistic, and include cross-feeding, antagonistic warfare via bacteriocins, local nutrient alteration or depletion, acidification of the micro-environment, substrate availability and others [32–37]. What counts essentially at an abstract level is that the micro-scale presence of one species alters the value of that same resource-growth unit complex for individuals of that same/other species, making it somewhat better or worse. Such altered susceptibilities to co-colonization *K*_*ij*_ (>1:  facilitation, <1:  competition) comprise a large potential spectrum (figure 1*c*), and for *N* = 2, form a 2-by-2 matrix which fully determines one of four possible outcomes between 2 species [38]: exclusion of 1, exclusion of 2, stable coexistence or bistability (figure 1*d*). Bistability means that one species or the other may be the sole winner in the system depending on initial frequency. However, the co-colonization interaction matrix in general is higher-dimensional *N* × *N*, when considering *N* possible ecological partners in the community. Singly and co-colonized units are assumed to produce an equal number of propagules, and mixed co-colonization units *I*_*ij*_ produce and transmit *i*/*j* propagules with probability 1/2 (a symmetry assumption that is relaxed in subsequent model extensions [31,39]).

Free growth units are replenished at rate *r* (in-flow), assumed equal to the natural decay rate (out-flow), keeping thus constant the carrying capacity of the system. Within each available micro-niche, microbes can grow for a mean duration of 1/*γ* time units, hence yielding a turnover rate of *m* = *r* + *γ*. The basic reproduction number *R*_0_ denotes how many new colonizations each colonized micro-niche will produce over its lifetime as occupied, if occurring in a totally colonization-free environment: *R*_0_ = *β*/*m*, and if *R*_0_ > 1 there is a globally stable endemic state with 1 − 1/*R*_0_ colonization prevalence, a classical feature of SIS models. If *R*_0_ < 1, the system is colonization-free.

### 3.2. Overarching dynamics of colonization and co-colonization

In the original model for *N* species, described in detail in [25], we track the variables *S*, *I*_*i*_ and *I*_*ij*_, that here refer to the proportion of uncolonized niches, those singly colonized by species *i* and those co-colonized by species *i* and *j*. The dynamics of colonization and co-colonization are given by the following equations:

3.1 $$\begin{array}{cc} & \frac{dS}{dt}=m(1-S)-S\sum_{\;j=1}^{N}F_{\;j},\;\;\;\;\;\;\\ & \frac{dI_{i}}{dt}=F_{i}S-mI_{i}-I_{i}\sum_{\;j}K_{ij}F_{\;j},\;1\leq i\leq N\\ \mathrm{and}\;\;\;\;\; & \frac{dI_{ij}}{dt}=I_{i}K_{ij}F_{\;j}-mI_{ij},\;\;\;\;1\leq i,j\leq N, \end{array}\}$$

where $F_{i}=\beta \left(I_{i}+\sum_{\;j=1}^{N}(1/2)(I_{ij}+I_{\;ji})\right)$ gives the force of propagation of species *i* in the system, summing the contribution of all micro-niches emitting propagules of that species. Denoting by $I=\sum_{i}I_{i}$ the total prevalence of single colonization, and $D=\sum_{i,j}I_{ij}$, the total prevalence of co-colonization, we have: *S* + *I* + *D* = 1. In this model in micro-niches co-colonized with different species *i* and *j*, the opportunity of each species to grow and be propagated is equal, thus represented with the 1/2 probability. In the system, the composite parameter *m* = *γ* + *r* represents the growth unit turnover rate, encapsulating both the clearance rate *γ* of each colonization episode, after which growth units become immediately available for new colonizations, and the natural replenishment rate of new universal units *r*. It is assumed that natural replenishment is balanced by natural out-flow rate *r* = *d*. Species interactions via local environmental modification are represented by the matrix *K*, where values *K*_*ij*_ above 1 indicate pairwise facilitation, while values of *K*_*ij*_ below 1 reflect inhibition between propagules of *i* and *j*, and where the order of colonizer and co-colonizer landing in a growth unit matters.

*From population structure to replicator dynamics.* The mathematical step that connects the population structure of the colonization with the frequency of individual species is time-scale separation [25,31], based on some similarity arguments for between-species traits.

In the most basic model [25], with variable co-colonization interaction coefficients *K*_*ij*_, the features of the full slow–fast approximation are summarized in table 1. Species similarity in traits *K*_*ij*_ means

$$K_{ij}=k+\epsilon \alpha_{ij},$$

with ϵ suitably small (e.g. the standard deviation of *K_ij_*) and *k* a benchmark, e.g. the mean of $K_{ij}\;:=\sum_{1\leq i,j\leq N}K_{ij}/N^{2}$. With this formulation, the full dynamics of the system can be decomposed into: *fast* dynamics, which are neutral and serve to equilibrate global population structure of colonization and co-colonization variables (see *S*, *I*, *D* in table 1), and *slow* dynamics, which are effectively the *N*-species frequency dynamics over the timescale τ=ϵt, captured by a replicator equation under some particular values of $\lambda_{i}^{\;j}$.

What is important in this separation of time-scales is that global variables from the fast dynamics stay constant during the slow dynamics, and can play a role in determining the magnitudes of the invasion fitnesses between species. During the slow dynamics $\lambda_{i}^{\;j}$ do not change, only species frequencies change obeying equation (2.2). This feature is robust also in extensions of the model [31,39].

### 3.3. How species traits inform invasion fitnesses $\lambda_{i}^{\;j}$

*Special case: species vary only in *K*_*ij*_.* In the model with variation only in co-colonization coefficients among species [25], species pairwise invasion fitness is given by

3.2 $$\lambda_{i}^{\;j}=\alpha_{\;ji}-\alpha_{\;jj}-\mu (\alpha_{ij}-\alpha_{\;ji}),$$

and is a direct function of the phenotypic variation in co-colonization interactions (the *α*_*ij*_’s), and of the ratio of single to co-colonization in the system:

$$\mu =\frac{I}{D}=\frac{1}{k(R_{0}-1)}.$$

What is key in this $\lambda_{i}^{\;j}$ is that the invasion performance of *i* into a *j*-*only* equilibrium depends on how much the resident entity *j* relatively favours the invader *i* over itself (trait difference *α*_*ji*_ − *α*_*jj*_ in $\lambda_{i}^{\;j}$) and also on the net benefit of *i* from transitions to mixed co-colonization with *j* (−*μ*(*α*_*ij*_ − *α*_*ji*_) term) [25,30]. This second term depends on relative availability of singly colonized micro-niches to co-colonize in the system, hence on the quantity *μ*.

Thus, there are two effects in this trait-based $\lambda_{i}^{\;j}$: (i) comparative advantage from non-self to self-interaction of the resident, and this is not dependent on co-colonization prevalence in the system and (ii) relative benefit from asymmetric investment in mixed co-colonization, and this decreases with prevalence of co-colonization in the system.

Notice that in this model, whether the species are competing or facilitating each other in their common growth on average (*k* → 0, or *k* large) has an effect on $\lambda_{i}^{\;j}$ via the parameter *μ*, which is an explicit decreasing function of *R*_0_ and *k*. The dependence on *μ* happens only for non-symmetric co-colonization interactions, i.e. only if *α*_*ji*_ ≠ *α*_*ij*_ and is stronger if the *α*′*s* are more anti-symmetric. For fixed *α*’s, the linear dependence of $\lambda_{i}^{\;j}$ on *μ*, implicating *R*_0_ and *k*, exemplifies an elegant contextual feedback on pairwise invasion fitness (*μ* high or low), which is key to understand analytically special cases of the replicator dynamics in these two extremes, and as a consequence, the limit cases of system invasion resistance [30].

*General case: species are more complex and vary along five fitness dimensions.* More generally, in a scenario when more traits are allowed to vary between species [31,39], the trait-based pairwise invasion fitness expression is more complex, but the same replicator dynamics can be derived. We outline in appendix A that the replicator dynamics (equation (2.2)) are general, and can be applied also to cases where species have different proliferation rates, clearance rates, and biases in local growth and priority effects; the pairwise invasion fitness in that case becomes a weighted average of the asymmetries along all phenotype axes (see eqn (2.22) in [31]).

Whatever their exact expression, together the $\lambda_{i}^{\;j}$ between all pairs drive collective dynamics in the system, and the definition of system invasion resistance via $Q=\bar{\lambda }$ remains robust.

## 4. Using the replicator equation to study system invasion fitness

### 4.1. Invasion resistance: system versus outsider species

**Figure 2.**
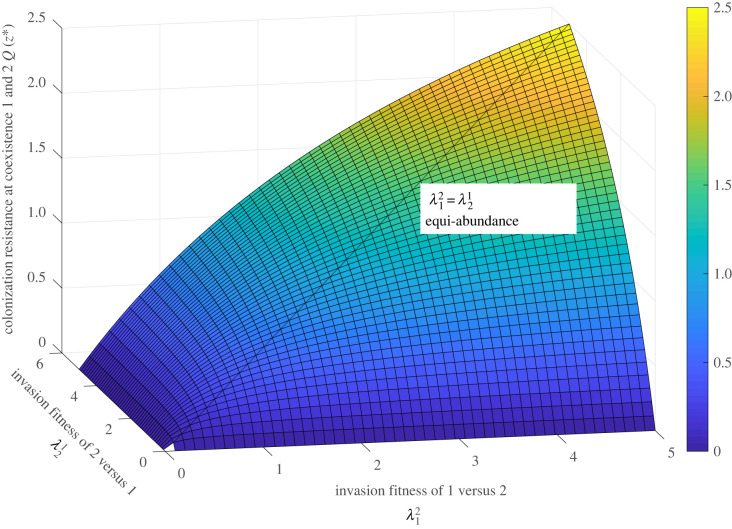
System invasion resistance *Q* in the *N* = 2 system with 2 species coexisting. Here we visualize a three-dimensional plot of *Q*(*x*, *y*) = *xy*/(*x* + *y*) as a function of mutual invasion fitnesses $x=\lambda_{1}^{2}$ and $y=\lambda_{2}^{1}$ when the system is at the coexistence equilibrium with species frequencies $\left(z_{1}^{*},z_{2}^{*})=(\lambda_{1}^{2}/(\lambda_{2}^{1}+\lambda_{1}^{2}),\lambda_{2}^{1}/(\lambda_{2}^{1}+\lambda_{1}^{2})\right)$. The maximum of *Q* is reached at $\lambda_{1}^{2}=\lambda_{2}^{1}$, i.e. for equiabundance of two resident species, and it increases with the absolute magnitude of the *λ*′s.

It is clear from equation (2.2), that to survive in the system, an individual/species must always ‘play games’ with co-occurring partners, and the payoff depends not only on an individual’s own (vector) strategy ($\lambda_{i}^{\;j},j=1,...,N$), but also on strategies and abundances of its opponents. Because *Q* sums products of frequencies, it is non-zero only when there are more than 1 species coexisting, and it is exactly zero when $\lambda_{\;j}^{k}=-\lambda_{k}^{\;j}$ for all pairs (*j*, *k*). In the simplest system with only two species, *N* = 2 (table 1), it is easy to see that at the coexistence steady state, *Q*(*z*_1_, *z*_2_) is positive and maximized for *z*_1_ = *z*_2_ = 1/2. Thus, a 2-species system is mostly protected against a third invader, when its two constituent members are equiabundant (figure 2).

Depending on how many species make up the resident species pool, *N*, and how they interact, one models a different system size and a different network of mutual invasion fitnesses (directed edges with precise magnitudes). This will generate system-specific frequency dynamics eventually leading up to a pruned community with *n* ≤ *N* species, and simultaneously the evolution of system resistance to invasion by outsiders (table 1).

**Figure 3.**
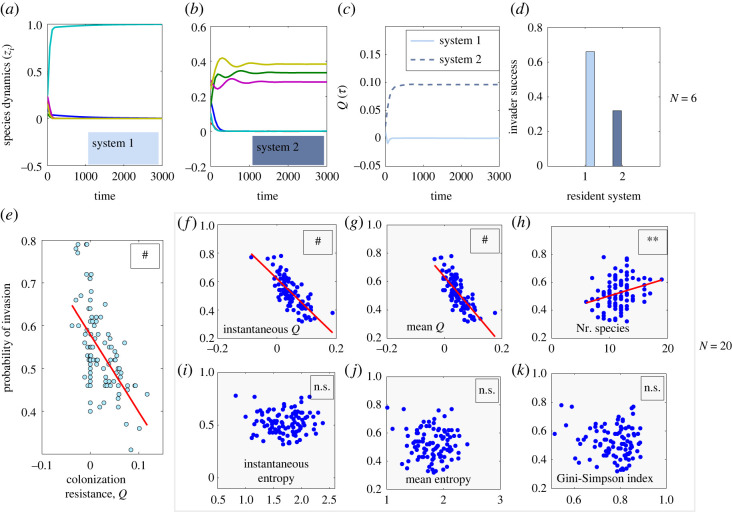
System invasion resistance, as defined by our model, and outsider invasion. In (*a*–*d*), we fixed the multispecies *K*_*ij*_ interactions in susceptibilities to co-colonization in two systems with *N* = 6, *β* = 1, *k* = 0.3, *R*_0_ = 2 but with different underlying species dynamics. We simulated 100 realizations of invasion by an outsider species, sampling its interaction traits from the same distribution as the resident $N(k,\epsilon^{2})$, and introducing it at the mid-point of the interval. We counted invasion successful if the invader grew from its low initial frequency (here 10^−4^). System 2 (higher *Q*) was superior in preventing invasion to system 1: experiencing only about 30% successful invasions compared to more than 60% successful invasions in system 1. In (*e*), we repeated the procedure for 100 randomly generated systems (blue dots) and examined system *Q* at invasion and invader success, finding a significant negative relationship between invasion resistance and invasibility (*p* < 10^−5^). The final outcome of invasion whether the invader ultimately coexists, or how original species are affected was not studied. In (*f*–*k*), we examined invasion success for *N* = 20. Simulating 100 random systems as in (*e*), we regressed outsider invasion probability on different system characteristics: *Q* (*p* < 10^−5^) in (*f*–*g*) and the number of species in the system (*p* < 0.01) in (*h*) were significant, while diversity indices (*i*–*k*) were not.

We tested several invasions numerically with model simulations, and how the success of an invader’s initial growth depends on system features. When comparing different multispecies systems in their resistance to invasion, for fixed parameters, we find that the higher the value of *Q*, the harder the invasion by outsider species (figure 3*a*–*g*). By contrast, we find no significant role of system diversity (Shannon, Gini–Simpson indices; figure 3*i*–*k*), but a positive effect of the number of species for invader success (figure 3*h*). The final outcome of any given invasion however, as captured in equation (2.4), besides system *Q*, depends also on other details of resident dynamics, and crucially on the traits of the invader, relative to the resident community, at the invasion time. This does not necessarily imply restrictions on cooperation or competition [40], but simply that the mean invasion fitness of the invader must exceed the mean invasion fitness of the resident system, whatever the path to satisfying such criterion may be.

### 4.2. Invading one system under different conditions: effect of global environmental perturbations

When the invasion fitnesses in the replicator equation have a mechanistic derivation (e.g. equation (3.2)) where a dependence with global quantities is immediately apparent (e.g. *μ* = 1/(*R*_0_ − 1)*k*), it is possible to study in some detail the system’s response to a change in such mean-field parameter. In the special case of a microbial ecosystem where invasion fitnesses are defined by (3.2), perturbations affecting global context, such as basic reproduction number *R*_0_ and mean interaction in co-colonization *k*, directly change the mutual invasion fitnesses $\lambda_{i}^{\;j}$ [25,30]. In highly diverse communities (many species *N* > 2, and/or more traits varying), effects of context-altering perturbations can be very complex, as we have shown in some cases [30,31,39].

However, among *N* species varying randomly only in co-colonization interaction phenotypes (susceptibilities *K*_*ij*_), a key modulator of coexistence regimes is the ratio of single- to co-colonization in the system, *μ* [30], decreasing with mean growth potential *R*_0_ and mean facilitation *k*. Perturbing these mean-field parameters can have predictable effects on invasion resistance of the system.

*How antibiotics alter colonization resistance of a microbial ecosystem:* *R*_0_ *effect.* One such perturbation example could be broad-spectrum antibiotics, leading to alteration of *R*_0_ in the system, likely reducing the colonization of all microbial species in the system, and shifting their internal competitive balance. Even when acting in the same way on system members, increasing their clearance rate or reducing proliferation, more antibiotics in our mechanistic model can drive the system from coexistence to competitive exclusion (see the case of *N* = 2 in figure 1*e*), hence toward *Q* = 0, promoting outsider invasion. Through our explicit $\lambda_{i}^{\;j}$ [25], one can clearly see that this *R*_0_ impact drastically varies, depending on the interaction network between species; for example it is stronger when *k* is lower, i.e. when competition is higher in the system (see electronic supplementary material, S3–S4). In particular, even when starting at the same baseline invasion fitnesses between two species (i.e. also the same coexistence scenario in terms of relative abundances), the stronger the mean competition in micro-scale co-colonization (the lower *k*), the more drastic will be the selection in the system induced by the antibiotic. Such explicit individualization of perturbation effects, based on underlying interactions, may be easily related to data and to context-dependence of outcomes in microbial consortia.

*How species facilitation alters colonization resistance of a microbial ecosystem:* *k* *effect.* In [30], we have shown in detail how in this model, *μ* = 1/((*R*_0_ − 1)*k*) acts as an axis of tunable contextual feedback on the multispecies dynamics: for standard normally distributed rescaled interaction strengths (*α*_*ij*_), when *μ* → ∞, and all else is kept fixed, the mutual invasibility network tends to contain mostly competitive exclusion edges, which makes more species coexist, but in unstable and oscillatory fashion, contrasting the regime of *μ* → 0 displaying mostly stable steady states. The extreme of *μ* → 0 can be obtained for increased facilitation between resident species (higher values of *k*), which typically would lead to higher invasion resistance of the system. This is similar to a result about cooperative microbiota obtained via simulations in other studies [40]. In our model, the phenomenon is entirely predictable analytically, and a similar effect could be obtained with amplifying *R*_0_, all else fixed. Conversely, decreasing *k*, hence decreasing facilitation, in the multispecies system the ratio of single- to co-colonization *μ* would increase, thereby promoting lower and oscillatory invasion resistance via fluctuating multispecies dynamics [30].

As the pivotal quantity in $\lambda_{i}^{\;j}$ is the product between *R*_0_ − 1 and *k*, an equivalent effect could be obtained by decreasing *R*_0_ in such a multispecies scenario. Lower overall colonization rate and higher *μ* could lead possibly to a fluctuating *Q* and could make a given system more vulnerable to opportunistic invasion, particularly when *Q* hits low or negative values, even if transiently. This seems to mimic what has been observed empirically, for example in individuals who following antibiotic therapy experienced domination of their gut microbiota by pathogenic *E. faecalis* or *C. difficile* bacteria, and succumbed to infection [15,41].

### 4.3. The role of pairwise invasion structures among *N* species

Above we considered a case of mechanistic knowledge of *λ* coefficients and their context-dependence, and how this would make them change with global parameters, leading to a predictable shift in system invasion resistance. What can we say about the species dynamics and invasion resistance of a system when the Λ matrix is quantitatively known but without explicit contextual drivers? How can we compare two systems that simply vary in their Λ matrix structure? We have previously sketched several canonical mutual invasion structures for the matrix of $\lambda_{i}^{\;j}$ in a system with *N* members [25], namely, invader-driven (equal columns); resident-driven (equal rows); symmetric ($\Lambda =\Lambda^{T}$); anti-symmetric ($\Lambda =-\Lambda^{T}$); and random.

In the special mechanistic case (3.2), these structures could be mapped to special assumptions about the co-colonization interaction matrix with *K*_*ij*_. For example, symmetric *K*, such that *K*_*ij*_ = *K*_*ji*_ = *K*_*ii*_ + *K*_*jj*_ − *k* leads to invader-driven Λ; whereas symmetric *K*, such that *K*_*ij*_ = *k* for *i* ≠ *j*, with the diagonal entries free to vary, leads to resident-driven Λ (see table 2 in [25] for more details).

However, there are in principle infinitely many possible microscopic *K*_*ij*_ or other trait combinations and model details, leading eventually to the same macroscopic $\lambda_{i}^{\;j}$ architecture. That is why next we revisit these Λ structures, more generally, from a top-down perspective, to explore their direct implications for invasion resistance in a completely closed multispecies ecosystem.

For symmetric mutual invasion ($\lambda_{i}^{\;j}=\lambda_{\;j}^{i}$), it can be shown that *Q* always increases (d*Q*/d*t* > 0), a case related to Fisher’s fundamental theorem [42] (for details see electronic supplementary material, section 4 in [25]). In such instance, cycles among species are impossible, and their dynamics converge typically to stable coexistence fixed points, and competitive exclusion is unlikely.

In the opposite extreme, for anti-symmetric mutual invasion ($\lambda_{i}^{\;j}=-\lambda_{\;j}^{i}$), the system colonization resistance is exactly zero over all time, *Q* = 0, corresponding to zero-sum games in evolutionary game-theoretic terms [43]. Dynamics in that case tend to a (structurally unstable) family of cycles around a centre, like in Lotka–Volterra prey–predator models [44], where an odd number of species coexist. More special cases, such as the species-centric *invader-driven* and *resident-driven* invasion, and explicit K–Λ matrix links are analysed in [25].

**Figure 4.**
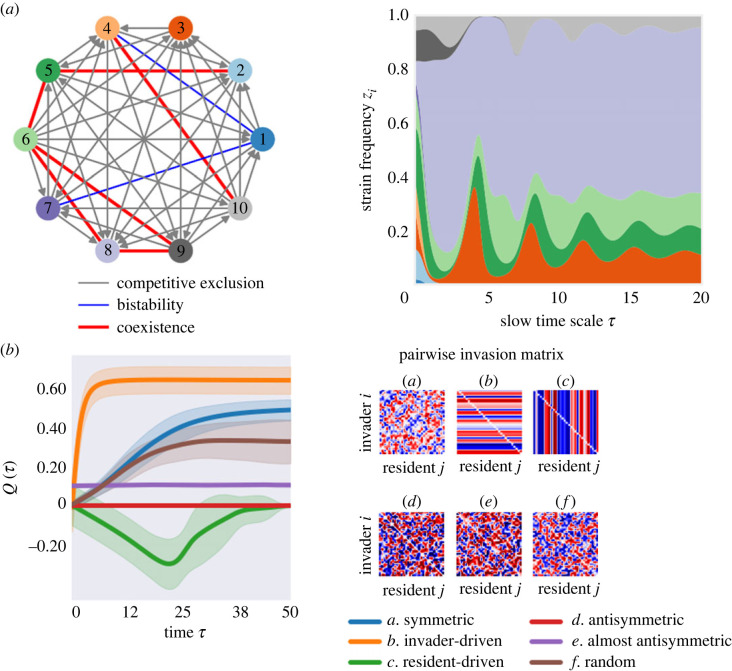
Mutual invasion structure of the species in the ecosystem, dynamics and system mean invasion fitness. (*a*) Illustration of a mutual invasion network between 10 species, whose structure and pairwise invasion fitness values lead to multispecies dynamics on the right (equation (2.2)), with ultimately five species coexisting. (*b*) Colonization resistance for special invasion structures. On the left, we plot *Q* dynamics (mean: lines, ±s.d.: shading) over 30 stochastic realizations for six cases of Λ between *N* = 30 species (shown on the right). For each case, random uniform $\lambda_{i}^{\;j}\in [-1,1]$, from the same distribution and range, are drawn and fed each into equation (2.2) (assuming Θ=1), which are then solved with random initial relative abundances *z*_*i*_(0), as in [25]. *Symmetric* Λ yields dynamics described by Fisher’s fundamental theorem, where mean invasion fitness always increases and stable coexistence is likely. *Invader-driven* Λ implies large coexistence potential among multiple species. *Resident-driven* Λ implies large potential for competitive exclusion, and favours a single species becoming the only persistent one of the community. *Antisymmetric* Λ makes *Q* ≡ 0, corresponding to zero-sum games among species and complex structurally unstable coexistence dynamics. *Almost-antisymmetric* Λ typically produces complex but more regular dynamics, for example limit cycles, leading to periodic *Q*. *Random* mutual invasion allows rich multispecies dynamics to unfold. Cases *a–c* can be obtained for three particular instances of symmetric co-colonization interactions matrix *K* [25].

Investigating community dynamics under these pairwise invasion architectures, we find that keeping all else equal, and starting from random initial frequencies, *Q* increases fastest over time in a system regulated by *invader-driven* mutual invasion (figure 4), i.e. where species are defined by their active invasiveness trait. This case typically leads to coexistence among several species and rarely to exclusion. In the *resident-driven* mutual invasion case, where species are defined by how they allow invasion by others, hence by their invasibility, dynamics tend most likely to competitive exclusion, i.e. toward *Q* = 0, but following an initial selection period during which systemic *Q* is negative.

A vast complexity of possible co-colonization interaction networks and emergent invasion architectures between *N* species may yield coexistence scenarios of more complex nature than fixed points, including multistable attractors, limit cycles and chaos [30]. Yet, in all these cases, the net invasion resistance that arises and changes as an adaptive trait of an evolving ecosystem can be mapped directly to the relative frequencies of constituent species. Thus, depending on how frequency dynamics unfold, subject to intrinsic or extrinsic drivers, *Q* dynamics can be higher or lower, monotonic or oscillatory, and feed back on the system, in a fully analytically explicit manner (table 1).

### 4.4. Invading one system at different times

**Figure 5.**
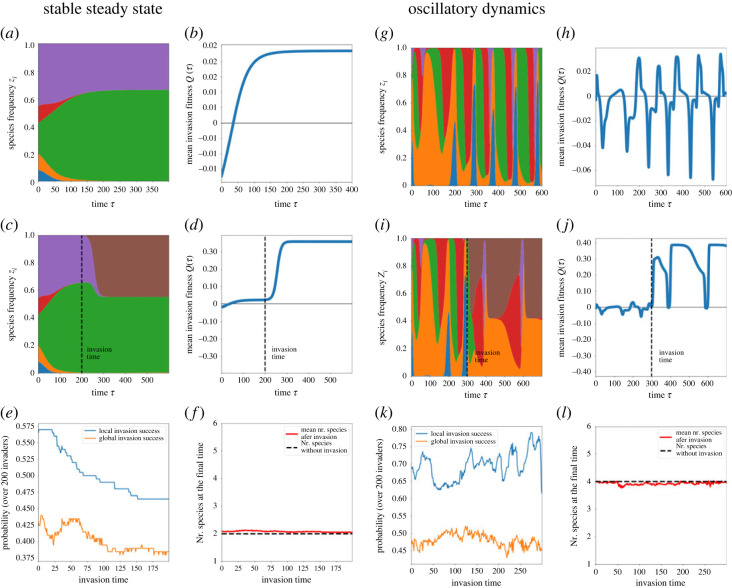
Two qualitatively different resident systems and sensitivity of invasion outcome to invasion timing. Here we fix two resident systems with *N* = 5 (*a–f* and *g–l*) and study the effect of invasion time over 200 random invaders. (*a*,*g*) The baseline dynamics of species frequencies in the absence of invasion (system with stable steady state and oscillatory dynamics, respectively). (*b,h*) The dynamics of mean invasion fitness of each system in the absence of invasion. (*c,i*) Example of species frequencies for a particular invader (in brown) and a particular invasion time (dashed line). (*d,j*) Corresponding mean invasion fitness dynamics for each system with invasion shown in (*c*,*i*). (*e,k*) Summary of many invasion outcomes as a function of invasion timing in the same system. We sample 200 invaders for each time point, simulate invasion starting from low initial frequency of 1%, and compute the proportion that results in initial growth (local invasion) and probability of ultimate persistence in the system (global success) using a dynamic horizon of *T* = 1000. In a system with monotonic $Q(\tau )=\bar{\lambda }$, mean invader success tends to a constant, whereas in the system with oscillatory $\bar{\lambda }$ invader success tends to oscillate. (*f,l*) One aspect of system outcome with and without invasion is the final number of coexisting species (here calculated as those above a given relative abundance threshold $z_{i}>1\%$ during the last 10% of the simulation time interval, and averaged over many different simulations). On the left, we observe that invasion may increase slightly number of coexisting species if invasion happens early in the course of community dynamics, when the system is far from equilibrium. If invasion happens closer to equilibrium, the qualitative number of final coexisting species tends to be the same, but with one of the species effectively replaced by the invader. On the right, we observe that invasion on average reduces diversity slightly below its originally expected level.

A corollary of this mathematical understanding of invasion is that following the dynamics of systemic mean invasion fitness $Q(\tau )=\bar{\lambda }$, the timing of invasion (by the same invader) will likely have a different success and effect on a given system, depending on the actual magnitude of *Q*. We performed simulations of invasion in two qualitatively different systems to illustrate this phenomenon: (i) in a system where multispecies coexistence tends to a stable fixed point (figure 5*a*,*b*) and (ii) in a system where underlying species coexistence is oscillatory (figure 5*g*,*h*). In figure 5*c*,*d*, we show one illustration of dynamics of a particular successful invader in multispecies system 1 (invader coloured in brown), and the corresponding dynamics of systemic invasion resistance *Q* after invasion. In figure 5*i*,*j*, we show one illustration of dynamics of another particular successful invader in multispecies system 2 (invader again coloured in brown), and the corresponding dynamics of systemic invasion resistance *Q* after this given invasion.

Then we repeat such invasion experiments over different invasion time points, and multiple random invaders. First, we confirm that temporal success of outsider invasion on average follows clearly the dynamics of *Q* of the underlying system: it tends to a constant when *Q* is monotonic (figure 5*e*), and oscillates periodically when *Q* is periodic (figure 5*k*). In particular, the less *Q* changes (i.e. when the underlying system tends to stable coexistence fixed points), the weaker the timing effect, except for a short transient whenever the resident community may be far from its equilibrium (figure 5*e*), and it may seem that the probability of successful invasion decreases with invasion time, until it saturates. Conversely, the more oscillatory invasion resistance *Q* is (i.e. the system is characterized by limit cycles), the stronger the timing effect on average, and invasion success can vary more along time (figure 5*k*), albeit being overall relatively higher.

These patterns seem to persist when computed both locally (invader growth after invasion) and globally (invader persistence after invasion), with the relative difference between the two correlated probabilities being about 20%. This suggests that on average the final fate of invaders may be quite predictable based on their initial invasion dynamics: if they invade, most likely they will persist (70–80% of invaders that do experience some growth in the system, will persist).

Our simulations also suggest that mean invasion fitness tends to increase after invasion (figure 5*d*,*j*), a plausible result when considering that a prerequisite for successful invasion is ${\bar{\lambda }}_{\mathrm{invader}}>Q={\bar{\lambda }}_{\mathrm{system}}$, so if an invader eventually manages to stay in the system it does so via augmenting systemic *Q*. Under stable coexistence, the successful invader tends to replace one species in a given niche—thereby not affecting final number of coexisting species *n* (figure 5*f*), but increasing the stability of this coexistence. In the oscillating case, such increase in stability comes from the successful invader more often decreasing the number of coexisting species (figure 5*g*).

### 4.5. Invading qualitatively different systems

**Figure 6.**
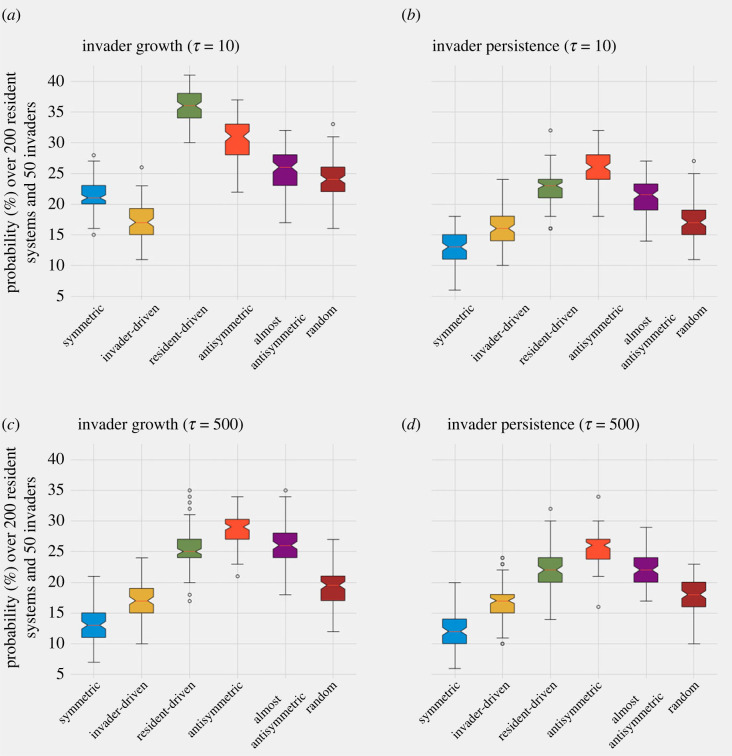
Mutual invasion structure of the resident microbial consortium and random invader success. We generated 200 random Λ matrix structures of size *N* = 5, with entry coefficients in the range [−1, 1], for each of six canonical architectures: symmetric, invader-driven, resident-driven, anti-symmetric, almost anti-symmetric and random. For each random invader (random uniform $\lambda_{\mathrm{invader}}^{\;j},\;j=1,...,5$), we simulated invasion in 200 systems of each type (Λ matrices), starting at the same initial frequency of the invader of 1% and equal relative frequencies of the resident five species totalling 99%. Over 200 systems, we computed the probability of outcome for each invader, summarized in the coloured boxplots, which show entire variation over 50 invaders for each type of Λ matrix. Systems were run for a time horizon of *T* = 1000. The invader was introduced at two timepoints: (*a,b*) early in the development of the system close to initial equiabundance (*τ* = 10), and (*c,d*) later in the development of the system, closer to its intrinsic equilibrium (*τ* = 500), which contains more signature of Λ-driven selection. Local invasion counts all those instances where the invader grew from its initial frequency at some point from introduction up to *T* = 1000. Global invasion counts all those instances where the random invader is still present in the system at final time *T* = 1000. The ranking observed between Λ structures can be very useful when engineering multispecies communities for the purpose of maximizing colonization resistance.

Random invader success can also be studied in given community contexts, fixing invasion time, but now varying systematically the underlying mutual invasion matrix Λ of the resident species (figure 6). In other words, how does the probability or random invader success depend on the ‘quality of the invasion game’ among the residents? For this, we performed 50 simulations of random outsider invasion, for 200 realizations of each canonical Λ structure (invader-driven, resident-driven, symmetric, anti-symmetric and random), and computed two indicators of successful invasion: (i) the probability of initial invader growth (local invasion) and (ii) the probability of final invader persistence in the system (global invasion).

As expected from our previous analysis [25] and the dynamics of *Q*(*τ*) in figure 4, we find that the mutual invasion structure in the resident community strongly impacts invader success. Importantly, the highest probability of invasion, both local and global, is obtained for the anti-symmetric invasion architecture of the resident species, yielding *Q* = 0 as the most favourable invasion context for outsiders. By contrast, the lowest probability of invader growth and invader persistence is found for symmetric, invader-driven and random mutual invasion of the resident species, indicating that these communities contain the least favourable (i.e. highest *Q*) colonization resistance for outsider invasion. This ranking is robust to random variation in the resident (200 different realizations of the same structure) and invader invasion coefficients (50 different invaders), pointing to a key signature of system stability and invasibility encoded precisely in the Λ matrix type. Although if introduced early (*τ* = 10), the probability of outsider species initial growth is lowest in a community characterized by invader-driven (row-only variation) Λ architecture (figure 6*a*), ultimate outsider persistence in the system (figure 6*b*), and in general both outsider growth and persistence for later invasions are minimized if the underlying system displays symmetric mutual invasion structure (blue boxplot in figure 6*c*,*d*).

Crucially, the ranking of these mutual invasion structures (Λ matrix types) suggests that the most resistant, externally stable communities are those whose constituent species display symmetric or hierarchical invader-driven pairwise invasion. Such Λ matrices, in our trait-based model, may be both obtained via special cases of symmetric co-colonization susceptibilities *K*_*ij*_ (see table 2 in [25]). This suggests that stronger collective coexistence may indeed arise when species pairs derive variable but symmetric benefits from shared resources.

Ultimately, the type of replicator dynamics critically depends on the species invasion matrix, whether the $\lambda_{i}^{\;j}$ are derived from particular biological traits and processes [25,31], or represented via qualitative structural constraints. While capturing all the realistic complexities and feedbacks between species traits, context, and systemic invasion resistance may be difficult at best, the conceptual perspective and results presented here should provide a new key to study system invasibility: precisely the architecture and magnitudes of mutual invasion coefficients among constituent species. This opens an avenue for exciting future developments in the study of invasion resistance using the replicator equation.

## 5. Discussion

Colonization resistance in microbiology [14] is closely linked with the theory of invasibility in ecology [11,45,46], long studied but rarely integrated between the disciplines. Despite our initial motivation, over the course of this study, it became increasingly clear that what we are advocating for goes in fact beyond microbiology, and constitutes an approach to study invasion resistance in multispecies systems via the replicator equation formalism. The microbiota analogy developed is useful to connect the dots from biological details to invasion fitness and replicator dynamics, and serves as an illustration of a very relevant context of potentially immediate applications, where this approach to study system invasibility can be adopted.

In the late 1950s, Elton argued that complex communities should be more resistant to invasion by new species [47]. Later May [3,4] showed that complex ecological communities tend to be less stable, i.e. in these systems it is harder to return to equilibria from small perturbations to existing species. Since then, the complexity–stability debate began, inspiring many investigations [11]. Crucial to study invasibility and stability of ecosystems have been simulations, using random interaction networks or varying their properties, e.g. the percentage of cooperative versus competitive links [48], or examining the relative interaction in invader versus within-resident community [40]. More recently, theoretical approaches are also invoking trait-based invasion fitness concepts [12].

Two notions of stability of a multispecies system, internal versus external, have been studied, with the latter receiving much less attention. It is external stability that relates most with invasion resistance. While the stabilizing versus destabilizing role of mutualism and cooperative interactions among species is still theoretically debated [48,49], as are diversity effects [50], recent work is attempting to reconcile Elton’s and May’s views [51] and seeking deeper syntheses. However until now, to our knowledge, no unifying formal analytical quantity has emerged for invasion resistance *per se*, that integrates species interactions along the competition–cooperation spectrum, their traits and resource availability.

In this paper, by calling attention on the replicator equation formalism, we propose a fresh conceptual framework to address invasibility, and in particular, to contribute to the formalization of colonization resistance concepts in microbiology [52]. Despite being the central equation of evolutionary game theory [8], the replicator equation is surprisingly little used in ecology or microbiology, in favour of the predominant use of generalized Lotka–Volterra frameworks for total abundances [5,7,49]. In our setup, we favour the use of the replicator equation because it precisely models the frequencies of multiple species, which are typically more readily available empirically, e.g. only relative abundances between (pseudo-) species of interest are most often empirically accessible via metagenomic sequencing data. In our model, chosen a ‘species’ resolution level, these relative abundances can be fully predicted via replicator dynamics.

Central to our replicator equation (equation (2.2)) is the measure of mutual invasion fitness between any two species, $\lambda_{i}^{\;j}$, and the various topologies of the matrix or contextual dependencies it generates. This higher-level trait inevitably arises as a function of lower-level traits and species asymmetries, including cooperation/competition (in our particular case co-colonization susceptibilities *K*_*ij*_), but also mean-field resource availability indicators (in our case *R*_0_, *k*)—dependencies which can be sometimes known or derived *a priori* from an underlying biological model. Within the replicator equation, these pairwise invasion fitnesses undergo selection and evolution in their own right following species frequency dynamics. Such evolution can be mapped to a collective mean system trait, $Q=\bar{\lambda }$, applicable to both equilibrium and non-equilibrium scenarios. This replicator framework unifies ecological, evolutionary dynamics and emergent system properties in multispecies communities. The same spirit of reconciling simplicity with complexity in functional biology and of improving predictability of models has also been advocated by others [53,54].

By illustrating a mechanistic path for such replicator equation, through an analogy with an epidemiological model [25], we simplify the complexity of multispecies dynamics, while including an abstract growth unit, integrating cooperation and competition in the same continuum via the tunable parameter *k*, and showing how the magnitude of the invasion fitness [24] can be bottom-up derived and trait-based. Other particular mechanistic or non-mechanistic realizations of such invasion fitness matrix between species could be studied in the future.

The whole-community behaviour exhibited in our model is a generic consequence of dynamic resource availability, similar to [55], where, in our case, abstract resources are amplified or diminished in value, by the simple co-presence of other ecological partners. In our closed system, essentially, species are simultaneously the ‘active’ entities, striving to survive, and the very ‘substrates’ upon which their life depends. There is no requirement for cooperative interactions in order for *Q* to evolve as a community-level function; only the possibility of species micro-scale co-occurrence, *k* > 0, is needed, coupled with the assumption that organisms transform their local environment in slightly different ways. Then it becomes another application of the basic notion that fitness is context-dependent [8,56–58].

Many theoretical phenomena, like those explored above and beyond, directly accessible via this formalism, could be empirically matched when comparing gradients in microbiota dynamics and composition, caused by antibiotics, diet or other stressors in multispecies communities, and relating such gradients to mathematical instances of classical replicator dynamics [30,44]. Empirically, these gradients could include variation across human or mammalian hosts of different age or conditions, or environments under the effect of nutrients and abiotic factors such as moisture, light, temperature (e.g. microbial consortia in soil, cheese rinds, natural biofilms).

Naturally, a conceptual and empirical challenge arises when wishing to access invasion or colonization resistance of a system. An idea can come from equation (2.4). Turning this equation around, one can see an indirect way of estimating $Q=\bar{\lambda }$ in a given system can bypass the precise knowledge of the *N*(*N* − 1)/2 pairwise invasion fitness coefficients of the constituent species, and just rely on a single observation of initial growth rate of any invader into the composite system (defined by *z*_*j*_), coupled with empirical observation of *N* pairwise invasion experiments of such invader versus each of the species (*j* = 1, ..., *N*) in the system. Further averaging of such experimental procedure over a few invaders can augment statistical power for estimation of *Q*. The framework should flexibly accommodate protocols of invasion experiments that consider all factors governing *Q*, namely species number and composition, their frequencies, and their time-dependence.

With theoretical extensions ongoing, to increase model scope and generality, for example adding space [59], or more trait dimensions for species differences [31,39], including growth and clearance biases besides interactions, possibly relevant to more biological scenarios [60], we show the backbone of the replicator equation formalism stays the same. This constitutes a big advantage as results on replicator dynamics [8] can be readily harnessed. The precise notion of invasion resistance, stemming from this model as mean invasion fitness of a community, can enable deeper study of the role of species interactions in the ecology of antibiotic resistance [61], of general community-level cohesion independently of cooperation or competition [55], and provide more precise quantifiable links with data, from both *in vitro* and *in vivo* systems [21,52,62].

In the light of increasing calls for models to better describe microbial consortia and predict system-level properties [53,54,63], here we outline a new path for explicit mathematical investigation of colonization resistance and mean invasion fitness in a microbial ecosystem. This replicator equation framework may help synthesize a new understanding of the ecology, development and evolution of host health and resilience to disease, mediated by microbiota. Its extended applications could also provide broader insights on invasibility [11,45,46,64] and invasion resistance in other multispecies ecosystems.

## Data Availability

Data and codes related to this study can be found on the Dryad Digital Repository: https://doi.org/10.5061/dryad.0k6djhb60 [65]. Supplementary files are provided in electronic supplementary material [66].

## Appendix A

**A.1. *N* colonizing species varying along five traits also follow replicator dynamics**

The more general colonization model for variation in other trait dimensions between similar species can be found in [31] where again the replicator equation with invasion fitnesses is derived mechanistically for the species frequency dynamics, using slow–fast dynamics arguments. Now, the *mechanistic* mutual invasion fitness $\lambda_{i}^{\;j}$ depends on *all* other traits, besides the matrix *K* = (*K*_*ij*_), describing micro-scale susceptibilities to co-colonization [25]. The five trait axes studied in [31,39] are:

(i) species-specific transmission/proliferation rates *β*_*i*_,
(ii) species-specific clearance rates in single colonization *γ*_*i*_,
(iii) pair-specific clearance rates in co-colonization (*γ*_*ij*_),
(iv) species onward propagation biases from mixed co-colonization, depending on their order of arrival ($p_{ij}^{i}\neq p_{ij}^{\;j}\neq 1/2$), also known as ‘priority effects’, and
(v) species pairwise susceptibilities to co-colonization *K*_*ij*_.

In this more complex scenario, the definition of ‘global context’ changes, as it may comprise also mean-field parameters in other traits, besides *R*_0_, *k* (relevant for the *K*_*ij*_-only model [25]). The *N* = 2-species system, in the case of 5-dimensional trait variation, is studied in [39], where the effect of the single to co-colonization ratio *μ* is shown to be very important, but this time influencing $\lambda_{i}^{\;j}$ in a nonlinear fashion. The dynamic invasion resistance of the *N*-species community emerges in this model again as a mean invasion fitness over all pairs in the system $Q=\bar{\lambda }$, dynamically shaped by their changing frequencies. This model generalization shows that studying system dynamics and evolution in terms of the mutual invasion coefficients between constituent species is robust and widely applicable.

## References

[1] Lotka AJ. 1920 Undamped oscillations derived from the law of mass action. J. Am. Chem. Soc. **42**, 1595-1599. (10.1021/ja01453a010)

[2] Volterra V. 1927 *Variazioni e fluttuazioni del numero d’individui in specie animali conviventi*, vol. 2. Societá anonima tipografica ‘Leonardo da Vinci’.

[3] May RM. 2019 Stability and complexity in model ecosystems, vol. 1. Princeton, NJ: Princeton University Press.

[4] May RM. 1972 Will a large complex system be stable? Nature **238**, 413-414. (10.1038/238413a0) 4559589

[5] Stein RR, Bucci V, Toussaint NC, Buffie CG, Rätsch G, Pamer EG, Sander C, Xavier JB. 2013 Ecological modeling from time-series inference: insight into dynamics and stability of intestinal microbiota. PLoS Comput. Biol. **9**, e1003388. (10.1371/journal.pcbi.1003388) 24348232 PMC3861043

[6] Faust K, Raes J. 2012 Microbial interactions: from networks to models. Nat. Rev. Microbiol. **10**, 538-550. (10.1038/nrmicro2832) 22796884

[7] Momeni B, Xie L, Shou W. 2017 Lotka-Volterra pairwise modeling fails to capture diverse pairwise microbial interactions. Elife **6**, e25051. (10.7554/eLife.25051) 28350295 PMC5469619

[8] Hofbauer J, Sigmund K. 2003 Evolutionary game dynamics. Bull. Am. Math. Soc. **40**, 479-519. (10.1090/S0273-0979-03-00988-1)

[9] Bomze IM. 1995 Lotka-Volterra equation and replicator dynamics: new issues in classification. Biol. Cybern. **72**, 447-453. (10.1007/BF00201420)

[10] Page KM, Nowak MA. 2002 Unifying evolutionary dynamics. J. Theor. Biol. **219**, 93-98. (10.1016/S0022-5193(02)93112-7) 12392978

[11] Levine JM, D’Antonio CM. 1999 Elton revisited: a review of evidence linking diversity and invasibility. Oikos **87**, 15-26. (10.2307/3546992)

[12] Hui C et al. 2021 Trait positions for elevated invasiveness in adaptive ecological networks. Biol. Invasions **23**, 1965-1985. (10.1007/s10530-021-02484-w)

[13] Bohnhoff M, Drake BL, Miller CP. 1954 Effect of streptomycin on susceptibility of intestinal tract to experimental Salmonella infection. Proc. Soc. Exp. Biol. Med. **86**, 132-137. (10.3181/00379727-86-21030) 13177610

[14] Lawley TD, Walker AW. 2013 Intestinal colonization resistance. Immunology **138**, 1-11. (10.1111/j.1365-2567.2012.03616.x) 23240815 PMC3533696

[15] Alonso CD, Treadway SB, Hanna DB, Huff CA, Neofytos D, Carroll KC, Marr KA. 2012 Epidemiology and outcomes of *Clostridium difficile* infections in hematopoietic stem cell transplant recipients. Clin. Infect. Dis. **54**, 1053-1063. (10.1093/cid/cir1035) 22412059 PMC3309884

[16] Owens Jr RC, Donskey CJ, Gaynes RP, Loo VG, Muto CA. 2008 Antimicrobial-associated risk factors for *Clostridium difficile* infection. Clin. Infect. Dis. **46**, S19-S31. (10.1086/521859) 18177218

[17] Jernberg C, Löfmark S, Edlund C, Jansson JK. 2007 Long-term ecological impacts of antibiotic administration on the human intestinal microbiota. ISME J. **1**, 56. (10.1038/ismej.2007.3) 18043614

[18] Carding S, Verbeke K, Vipond DT, Corfe BM, Owen LJ. 2015 Dysbiosis of the gut microbiota in disease. Microb. Ecol. Health Dis. **26**, 26191. (10.3402/mehd.v26.26191) 25651997 PMC4315779

[19] Van Nood E et al. 2013 Duodenal infusion of donor feces for recurrent *Clostridium difficile*. N. Engl. J. Med. **368**, 407-415. (10.1056/NEJMoa1205037) 23323867

[20] Lozupone CA, Stombaugh JI, Gordon JI, Jansson JK, Knight R. 2012 Diversity, stability and resilience of the human gut microbiota. Nature **489**, 220-230. (10.1038/nature11550) 22972295 PMC3577372

[21] Sousa A, Frazão N, Ramiro RS, Gordo I. 2017 Evolution of commensal bacteria in the intestinal tract of mice. Curr. Opin. Microbiol. **38**, 114-121. (10.1016/j.mib.2017.05.007) 28591676

[22] Oliveira RA, Ng KM, Correia MB, Cabral V, Shi H, Sonnenburg JL, Huang KC, Xavier KB. 2020 Klebsiella michiganensis transmission enhances resistance to Enterobacteriaceae gut invasion by nutrition competition. Nat. Microbiol. **5**, 630-641. (10.1038/s41564-019-0658-4) 31959968

[23] Kim S, Covington A, Pamer EG. 2017 The intestinal microbiota: antibiotics, colonization resistance, and enteric pathogens. Immunol. Rev. **279**, 90-105. (10.1111/imr.12563) 28856737 PMC6026851

[24] Geritz SA, Kisdi E, Meszé NAG, Metz JA. 1998 Evolutionarily singular strategies and the adaptive growth and branching of the evolutionary tree. Evol. Ecol. **12**, 35-57. (10.1023/A:1006554906681)

[25] Madec S, Gjini E. 2020 Predicting N-strain coexistence from co-colonization interactions: epidemiology meets ecology and the replicator equation. Bull. Math. Biol. **82**, 142. (10.1007/s11538-020-00816-w) 33119836 PMC7595998

[26] Hsu SB, Hubbell S, Waltman P. 1978 Competing predators. SIAM J. Appl. Math. **35**, 617-625. (10.1137/0135051)

[27] Friedman J, Higgins LM, Gore J. 2017 Community structure follows simple assembly rules in microbial microcosms. Nat. Ecol. Evol. **1**, 0109. (10.1038/s41559-017-0109) 28812687

[28] Wright ES, Gupta R, Vetsigian KH. 2021 Multi-stable bacterial communities exhibit extreme sensitivity to initial conditions. FEMS Microbiol. Ecol. **97**, fiab073. (10.1093/femsec/fiab073) 34021563

[29] Remien CH, Eckwright MJ, Ridenhour BJ. 2021 Structural identifiability of the generalized Lotka–Volterra model for microbiome studies. R. Soc. Open Sci. **8**, 201378. (10.1098/rsos.201378) 34295510 PMC8292772

[30] Gjini E, Madec S. 2021 The ratio of single to co-colonization is key to complexity in interacting systems with multiple strains. Ecol. Evol. **11**, 8456-8474. (10.1002/ece3.7259) 34257910 PMC8258234

[31] Le TMT, Gjini E, Madec S. 2023 Quasi-neutral dynamics in a coinfection system with *N* strains and asymmetries along multiple traits. J. Math. Biol. **87**, 48. (10.1007/s00285-023-01977-7) 37640832

[32] Faith JJ et al. 2013 The long-term stability of the human gut microbiota. Science **341**, 1237439. (10.1126/science.1237439) 23828941 PMC3791589

[33] Gralka M, Szabo R, Stocker R, Cordero OX. 2020 Trophic interactions and the drivers of microbial community assembly. Curr. Biol. **30**, R1176-R1188. (10.1016/j.cub.2020.08.007) 33022263

[34] Granato ET, Meiller-Legrand TA, Foster KR. 2019 The evolution and ecology of bacterial warfare. Curr. Biol. **29**, R521-R537. (10.1016/j.cub.2019.04.024) 31163166

[35] Walker AW, Duncan SH, McWilliam Leitch EC, Child MW, Flint HJ. 2005 pH and peptide supply can radically alter bacterial populations and short-chain fatty acid ratios within microbial communities from the human colon. Appl. Environ. Microbiol. **71**, 3692-3700. (10.1128/AEM.71.7.3692-3700.2005) 16000778 PMC1169066

[36] Cotter PD, Ross RP, Hill C. 2013 Bacteriocins—a viable alternative to antibiotics? Nat. Rev. Microbiol. **11**, 95-105. (10.1038/nrmicro2937) 23268227

[37] Pande S, Merker H, Bohl K, Reichelt M, Schuster S, De Figueiredo LF, Kaleta C, Kost C. 2014 Fitness and stability of obligate cross-feeding interactions that emerge upon gene loss in bacteria. ISME J. **8**, 953-962. (10.1038/ismej.2013.211) 24285359 PMC3996690

[38] Gjini E, Madec S. 2017 A slow-fast dynamic decomposition links neutral and non-neutral coexistence in interacting multi-strain pathogens. Theor. Ecol. **10**, 129-141. (10.1007/s12080-016-0320-1)

[39] Le TMT, Madec S, Gjini E. 2022 Disentangling how multiple traits drive 2 strain frequencies in SIS dynamics with coinfection. J. Theor. Biol. **538**, 111041. (10.1016/j.jtbi.2022.111041) 35114194

[40] Kurkjian HM, Akbari MJ, Momeni B. 2021 The impact of interactions on invasion and colonization resistance in microbial communities. PLoS Comput. Biol. **17**, e1008643. (10.1371/journal.pcbi.1008643) 33481772 PMC7857599

[41] Ubeda C et al. 2010 Vancomycin-resistant Enterococcus domination of intestinal microbiota is enabled by antibiotic treatment in mice and precedes bloodstream invasion in humans. J. Clin. Invest. **120**, 4332-4341. (10.1172/JCI43918) 21099116 PMC2993598

[42] Fisher RA. 1958 The genetical theory of natural selection. Oxford, UK: Clarendon Press.

[43] Chawanya T, Tokita K. 2002 Large-dimensional replicator equations with antisymmetric random interactions. J. Phys. Soc. Jpn. **71**, 429-431. (10.1143/JPSJ.71.429)

[44] Allesina S, Levine JM. 2011 A competitive network theory of species diversity. Proc. Natl Acad. Sci. USA **108**, 5638-5642. (10.1073/pnas.1014428108) 21415368 PMC3078357

[45] Elton CS. 2020 The ecology of invasions by animals and plants. Berlin, Germany: Springer Nature.

[46] Davis MA, Grime JP, Thompson K. 2000 Fluctuating resources in plant communities: a general theory of invasibility. J. Ecol. **88**, 528-534. (10.1046/j.1365-2745.2000.00473.x)

[47] Kitching RL. 2010 A world of thought: the ecology of invasions by animals and plants and Charles Elton’s life’s work. In Fifty years of invasion ecology: the legacy of Charles Elton (ed. DM Richardson), pp. 1-10. Chichester, UK: Wiley-Blackwell. (10.1002/9781444329988.ch1)

[48] Qian JJ, Akçay E. 2020 The balance of interaction types determines the assembly and stability of ecological communities. Nat. Ecol. Evol. **4**, 356-365. (10.1038/s41559-020-1121-x) 32094535

[49] Coyte KZ, Schluter J, Foster KR. 2015 The ecology of the microbiome: networks, competition, and stability. Science **350**, 663-666. (10.1126/science.aad2602) 26542567

[50] Fridley JD, Stachowicz JJ, Naeem S, Sax D, Seabloom E, Smith M, Stohlgren T, Tilman D, Holle BV. 2007 The invasion paradox: reconciling pattern and process in species invasions. Ecology **88**, 3-17. (10.1890/0012-9658(2007)88[3:TIPRPA]2.0.CO;2) 17489447

[51] Hui C, Richardson DM. 2019 How to invade an ecological network. Trends Ecol. Evol. **34**, 121-131. (10.1016/j.tree.2018.11.003) 30514581

[52] Boureau L, Hartmann T, Karjalainen T, Rowland I, Wilkinson MHF. 2000 Models to study colonisation and colonisation resistance. Microb. Ecol. Health Dis. **12**, 247-258. (10.1080/089106000750060503)

[53] Bergelson J, Kreitman M, Petrov DA, Sanchez A, Tikhonov M. 2021 Functional biology in its natural context: a search for emergent simplicity. Elife **10**, e67646. (10.7554/eLife.67646) 34096867 PMC8184206

[54] Widder S et al. 2016 Challenges in microbial ecology: building predictive understanding of community function and dynamics. ISME J. **10**, 2557-2568. (10.1038/ismej.2016.45) 27022995 PMC5113837

[55] Tikhonov M. 2016 Community-level cohesion without cooperation. Elife **5**, e15747. (10.7554/eLife.15747) 27310530 PMC4946899

[56] Hay ME, Parker JD, Burkepile DE, Caudill CC, Wilson AE, Hallinan ZP, Chequer AD. 2004 Mutualisms and aquatic community structure: the enemy of my enemy is my friend. Annu. Rev. Ecol. Evol. Syst. **35**, 175-197. (10.1146/annurev.ecolsys.34.011802.132357)

[57] McGill BJ, Enquist BJ, Weiher E, Westoby M. 2006 Rebuilding community ecology from functional traits. Trends Ecol. Evol. **21**, 178-185. (10.1016/j.tree.2006.02.002) 16701083

[58] Ribeck N, Lenski RE. 2015 Modeling and quantifying frequency-dependent fitness in microbial populations with cross-feeding interactions. Evolution **69**, 1313-1320. (10.1111/evo.12645) 25787308

[59] Le TMT, Madec S. 2023 Spatiotemporal evolution of coinfection dynamics: a reaction–diffusion model. J. Dyn. Diff. Equat. 10.1007/s10884-023-10285-z)

[60] Schluter J, Foster KR. 2012 The evolution of mutualism in gut microbiota via host epithelial selection. PLoS Biol. **10**, e1001424. (10.1371/journal.pbio.1001424) 23185130 PMC3502499

[61] Bottery MJ, Pitchford JW, Friman VP. 2021 Ecology and evolution of antimicrobial resistance in bacterial communities. ISME J. **15**, 939-948. (10.1038/s41396-020-00832-7) 33219299 PMC8115348

[62] Ratzke C, Barrere J, Gore J. 2020 Strength of species interactions determines biodiversity and stability in microbial communities. Nat. Ecol. Evol. **4**, 376-383. (10.1038/s41559-020-1099-4) 32042124

[63] Borenstein E. 2012 Computational systems biology and in silico modeling of the human microbiome. Brief. Bioinform. **13**, 769-780. (10.1093/bib/bbs022) 22589385

[64] Davis MA, Pelsor M. 2001 Experimental support for a resource-based mechanistic model of invasibility. Ecol. Lett. **4**, 421-428. (10.1046/j.1461-0248.2001.00246.x)

[65] Gjini E, Madec S. 2023 Data from: Towards a mathematical understanding of invasion resistance in multispecies communities. Dryad Digital Repository. (10.5061/dryad.0k6djhb60) PMC10646464 38026034

[66] Gjini E, Madec S. 2023 Towards a mathematical understanding of invasion resistance in multispecies communities. Figshare. (10.6084/m9.figshare.c.6915884) PMC10646464 38026034

